# Entrance‐Site Ablation in Atrial Tachycardia Involving Epicardial Conduction: A Case Series

**DOI:** 10.1002/joa3.70390

**Published:** 2026-06-16

**Authors:** Yuichiro Sakamoto, Yuko Uemura, Ryo Yamaguchi, Hirokazu Naganawa, Daisuke Yoshimoto, Takahiko Suzuki

**Affiliations:** ^1^ Department of Cardiovascular Medicine Toyohashi Heart Center Toyohashi Aichi Japan

**Keywords:** atrial tachycardia, entrainment, epicardial conduction

## Abstract

**Background:**

Treatment of atrial tachycardia (AT) involving epicardial conduction remains challenging. While endocardial exit sites can serve as targets for radiofrequency (RF) ablation, this approach is sometimes ineffective and may result in treatment failure.

**Methods:**

This retrospective study systematically investigated entrance‐site ablation for 10 ATs involving epicardial conduction in nine patients who were unresponsive to initial exit‐site ablation. The entrance site—identified using high‐resolution endocardial mapping and entrainment as the most downstream site where the postpacing interval closely approximated the AT cycle length (ATCL)—was targeted for RF ablation.

**Results:**

The median ATCL was 328 ms (289–356). The missing activation time was 140 ms (93–157), corresponding to 42% (35–47) of the ATCL. Possible epicardial conduction inferred from entrainment findings was attributed to the vein/ligament of Marshall in four ATs, the septopulmonary bundle in four, and combinations involving the Bachmann bundle in two. RF ablation at the entrance site terminated four ATs with a single RF application, while in the remaining six, additional lesions with progressive cycle‐length prolongation achieved termination. Noninducibility was confirmed in all patients.

**Conclusions:**

Systematic identification and ablation of the entrance site of epicardial conduction using detailed entrainment mapping provide a practical and effective strategy for managing ATs involving epicardial conduction, particularly when conventional exit‐site ablation is insufficient.

## Graphical abstract

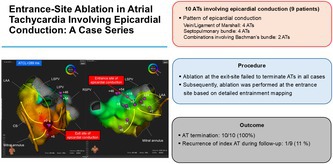

## 1 Introduction

Atrial tachycardia (AT) frequently emerges following atrial fibrillation (AF) ablation or left atriotomy [1]. Although high‐resolution mapping has clarified various AT circuits, those involving epicardial conduction pose additional challenges in both circuit interpretation and treatment. In such AT involving epicardial conduction, the endocardial exit site may appear focal and often serves as a radiofrequency (RF) ablation target; however, this approach can sometimes be ineffective, leading to treatment failure [2, 3, 4]. The aim of this study was to investigate the utility of performing detailed entrainment to localize the entrance site and guide ablation at that location in patients with AT involving epicardial conduction.

## 2 Methods

### 2.1 Patient Selection

Between June 2016 and June 2025, we screened patients who underwent ablation for AF and/or AT at the Toyohashi Heart Center. Nine patients (10 ATs) diagnosed with AT involving epicardial conduction were included. These were cases in which detailed entrainment mapping during the procedure suggested the presence of both endocardial exit and possible epicardial entrance sites, and in which initial ablation at the exit site failed to terminate the tachycardia. ATs involving epicardial conduction via the coronary sinus (CS) musculature were excluded because of their high prevalence in patients with a history of mitral isthmus (MI) ablation and the fact that ablation from within the CS is generally considered the standard approach in such cases. All procedures were performed in accordance with the ethical standards of the institutional and/or national research committee and the 1964 Declaration of Helsinki and its later amendments or comparable ethical standards. All patients provided written informed consent, and the study was approved by the Toyohashi Heart Center ethics committee. Oral anticoagulation was administered for at least one month before and continued throughout the procedure. Antiarrhythmic drugs were discontinued at least five half‐lives before the procedure. No patients in this study were taking amiodarone. Transesophageal echocardiography and computed tomography were performed to exclude atrial thrombus and visualize the left atrium (LA), pulmonary veins (PVs), and the CS anatomy.

### 2.2 Electrophysiological Study

All procedures were performed under deep sedation with fentanyl and dexmedetomidine. A 20‐pole catheter (2 mm‐interelectrode spacing, edge‐to‐edge) was inserted via the jugular vein into the CS. Heparin was administered to maintain an activated clotting time of > 300 s. Two transseptal sheaths were inserted into the LA via transseptal puncture performed under intracardiac echocardiography (AcuNav) guidance. Intracardiac electrograms (EGMs) and surface twelve‐lead electrocardiogram (ECG) were continuously monitored and recorded (Bard‐Lab‐System‐Pro). Bipolar and unipolar electrograms were filtered at 30–500 Hz and 1–300 Hz, respectively. A three‐dimensional electroanatomical map of the LA and PVs was constructed using one of the following high‐resolution mapping catheters: the Orion catheter with Rhythmia system (Boston Scientific), the PentaRay catheter with CARTO3 system (Biosense Webster), the OctaRay catheter with CARTO3 system (Biosense Webster), or the HD Grid catheter with EnSite X (Abbott) during sustained ATs. Scar was defined as bipolar voltage < 0.03 mV in the CARTO and Rhythmia systems and < 0.05 mV in the EnSite system. Voltage display settings were adjusted when necessary to optimize visualization of low‐amplitude electrograms.

### 2.3 Diagnosis of AT Involving Epicardial Conduction, Identification of the Entrance Site, and Ablation Strategy

We adopted a predefined, stepwise, entrainment‐guided entrance‐site ablation strategy for ATs involving epicardial conduction. After construction of a high‐resolution activation map, AT involving epicardial conduction was diagnosed if all of the following criteria were met:

1. The total endocardial activation time did not cover the entire AT cycle length (ATCL).
2. The activation map demonstrated an apparent endocardial “exit site” resembling focal AT.
3. Entrainment pacing showed a postpacing interval (PPI) closely matching the ATCL (≤ 20 ms) at multiple sites remote from the endocardial exit site.

In this study, the endocardial exit site was defined based on focal or breakout activation patterns on high‐resolution activation mapping, whereas the entrance site of epicardial conduction was identified using entrainment mapping criteria. Once an AT was diagnosed as involving epicardial conduction, detailed entrainment mapping was performed in a systematic manner along the direction of endocardial activation propagation originating from the exit site, as identified on the activation map. Pacing sites were sequentially selected downstream along this propagation path, and PPI was assessed at each site. During entrainment pacing, the pacing output was set at 5 V with a pulse width of 1.0 ms to minimize the risk of far‐field capture. If consistent local capture could not be achieved at a given site, pacing was performed from an adjacent endocardial site rather than increasing the pacing output. When far‐field components were suspected, electrograms were interpreted primarily based on local activation timing and spatial continuity on high‐resolution activation mapping. Sites demonstrating a PPI closely matching the ATCL were considered to be within the reentrant circuit. Entrainment was continued progressively downstream until either a transition point was identified where the PPI no longer matched the ATCL (> 20 ms), or the pacing site reached an anatomical boundary or the edge of an endocardial scar beyond which further downstream entrainment was not feasible despite preserved PPI–ATCL concordance. The most downstream site at which the PPI remained equal to the ATCL was presumed to represent the entrance site of the epicardial conduction. Although orthodromic capture of the exit site during pacing from the presumed entrance site was observed in several cases, this maneuver was not systematically performed in all patients.

RF energy at 35–40 W with an irrigated‐tip ablation catheter was initially delivered for 30–60 s at the exit site rather than the entrance site of the epicardial conduction. If the ablation site was adjacent to the esophagus, an esophageal temperature probe was inserted, and the RF application was terminated when the esophageal temperature reached 39.5°C. For ATs suspected of involving the vein/ligament of Marshall (VOM/LOM), empirical RF applications were additionally performed along the left atrial appendage (LAA) ridge near the level of the left inferior pulmonary vein (LIPV). After RF applications failed to terminate AT at the exit site, RF energy at 30–45 W was delivered for 30–60 s to the entrance site of the epicardial conduction, as identified by entrainment mapping. When the initial RF application prolonged the ATCL but failed to terminate the AT, additional RF applications were performed at slightly shifted sites surrounding the initial lesion.

After termination of AT, pacing from sites adjacent to the successful ablation lesion was performed to confirm the absence of breakout at the previously identified exit site. Programmed atrial stimulation was subsequently performed to assess inducibility. Acute procedural success was defined as termination of AT and non‐inducibility.

### 2.4 Follow‐Up

All patients were followed with ECGs at 1, 3, 6, 9, and 12 months and every 6 months thereafter. Holter recordings (24 h, 5 days, or 2 weeks) were performed at 3, 6, and 12 months. Event recording was performed if patients reported symptoms suspicious of arrhythmia recurrence. Antiarrhythmic drugs were discontinued within the initial 3‐month blanking period. Arrhythmia recurrence was defined as any episode of AT/AF lasting more than 30 s after the blanking period.

### 2.5 Statistical Analysis

Continuous variables are expressed as mean ± standard deviation (SD) when normally distributed, and as median with interquartile range (IQR) when not. Categorical variables are presented as numbers and percentages. The Wilcoxon signed‐rank test was used to compare paired non‐normally distributed continuous variables. Categorical variables were compared using Fisher's exact test when appropriate. Statistical significance was assumed at *p* < 0.05. All statistical analyses were performed using EZR (Saitama Medical Center, Jichi Medical University, Saitama, Japan), which is a graphical user interface for R (The R Foundation for Statistical Computing, Vienna, Austria) [5].

## 3 Results

### 3.1 Characteristics of Study Patients

Characteristics of study patients are shown in Table 1. The median number of prior procedures was 2 (1, 2). The indication for ablation was AT in five patients, and both AT and AF in the remaining four.

**TABLE 1:** Baseline characteristics of study patients (9 patients).

Characteristic	Value	
Age, years	71 ± 11	
Sex, female	5	56%
BMI, kg/m^2^	22.6 ± 4.1	
Hypertension	4	44%
CHA_2_DS_2_‐VASc score	2	(2–5)
Structural heart disease	1	11%
Echocardiography		
LA diameter, mm	43 ± 7	
LV ejection fraction, %	54 ± 14	
Indication for this procedure		
AT	5	56%
AF and AT	4	44%
Prior ablation procedures	8	89%
Number of prior procedures	2	(1, 2)
Details of the prior procedure (s)		
PV isolation	8	100%
SVC isolation	3	38%
GP ablation	3	38%
CFAE ablation	2	25%
LA roof line	2	25%
Posterior wall isolation	1	13%
CTI line	4	50%
Details of this procedure		
Initial or repeat PV isolation	2	22%
CFAE ablation	3	33%
LA roof line	5	56%
MI line	5	56%
Posterior wall isolation	3	33%
CTI line	1	11%
Ablation of AT involving epicardial conduction	9	100%

*Note:* Values are expressed as mean ± standard deviation for normally distributed continuous variables, and as median (interquartile range) for non‐normally distributed variables. Categorical variables are presented as a number (percentage). Details of prior procedures are shown for the eight patients with a history of previous ablation. Percentages in this section are calculated using *n* = 8 as the denominator.

Abbreviations: AF, atrial fibrillation; AT, atrial tachycardia; BMI, body mass index; CFAE, complex fractionated atrial electrograms; CTI, Cavo‐tricuspid isthmus; GP, ganglionated plexus; LA, left atrium; LV, left ventricle; MI, mitral isthmus; PV, pulmonary vein; SVC, superior vena cava.

### 3.2 Ablation to the Entrance Site of Epicardial Conduction in Patients With ATs

**FIGURE 1:**
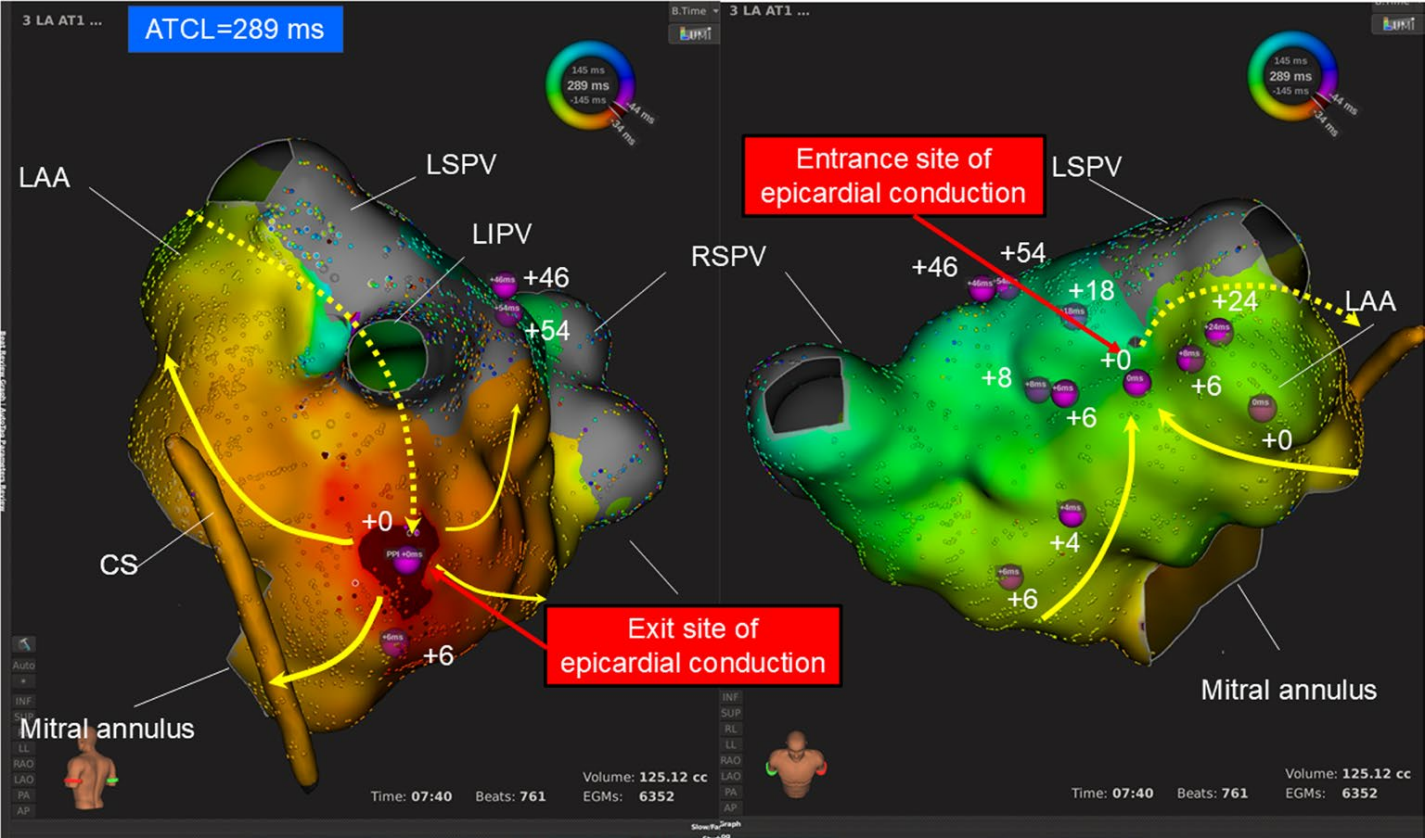
Activation map and entrainment findings in Patient 1. Purple tags indicate the difference between PPI and ATCL, with values shown as “+xx (ms)”. Yellow arrows show endocardial activation of the AT. Based on entrainment findings, the possible epicardial channel of the AT is VOM/LOM (yellow dash arrow). AT = atrial tachycardia; ATCL = atrial tachycardia cycle length; CS = coronary sinus; LA = left atrium; LAA = left atrial appendage; LIPV = left inferior pulmonary vein; LOM = ligament of Marshall; LSPV = left superior pulmonary vein; PPI = post pacing interval; RF = radiofrequency; RSPV = right superior pulmonary vein; RIPV = right inferior pulmonary vein; VOM = vein of Marshall.

Patient 1 represented a typical example of AT involving the VOM/LOM (Figure 1 and Video S1). Although the activation map initially suggested a focal AT originating near the lower aspect of the LIPV, PPIs obtained from multiple points on the LA septum and anterior wall were nearly equal to ATCL. Consequently, this finding was interpreted as macroreentrant AT rather than purely focal AT. Entrainment mapping demonstrated that the furthest downstream site with a PPI equal to the ATCL was located near the top of the LAA ridge, serving as the entrance of the epicardial conduction via the VOM/LOM for the reentrant circuit. RF applications at the exit site and along the LAA ridge at the level of the LIPV did not result in cycle length prolongation or termination of the AT. However, RF applications at the entrance site resulted in AT termination after 4.7 s.

**FIGURE 2:**
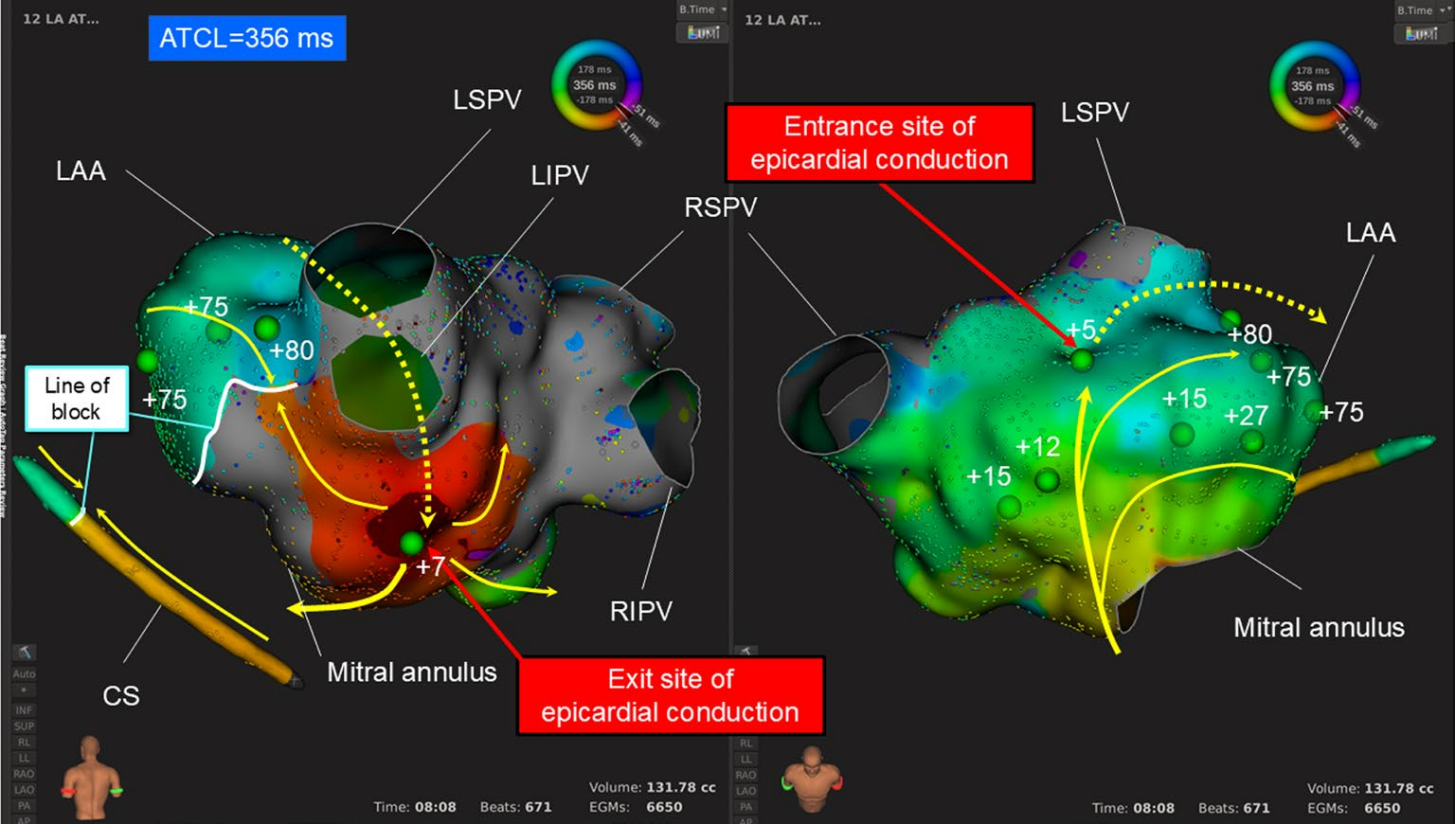
Activation map and entrainment findings in Patient 2. Green tags indicate the difference between PPI and ATCL, with values shown as “+xx (ms)”. Yellow arrows show endocardial activation of the AT. Based on entrainment findings, the possible epicardial channel of the AT is VOM/LOM (yellow dashed arrow). Abbreviations as in Figure 1.

Patient 2 was another example of AT involving the VOM/LOM (Figure 2 and Video S2). Activation map showed an apparently focal AT near the lower portion of the LIPV, yet entrainment at the LA septum and anterior wall demonstrated that the PPI closely matched the ATCL, leading to the diagnosis of macroreentrant AT. The critical site was again at the top of the LAA ridge, with the VOM/LOM forming an epicardial pathway as in Patient 1. RF applications at the exit site had no effect on the AT cycle length or termination. The ridge at the level of the LIPV had already been extensively ablated to create the lateral MI block. RF applications at the entrance site led to AT termination after 25.3 s.

**FIGURE 3:**
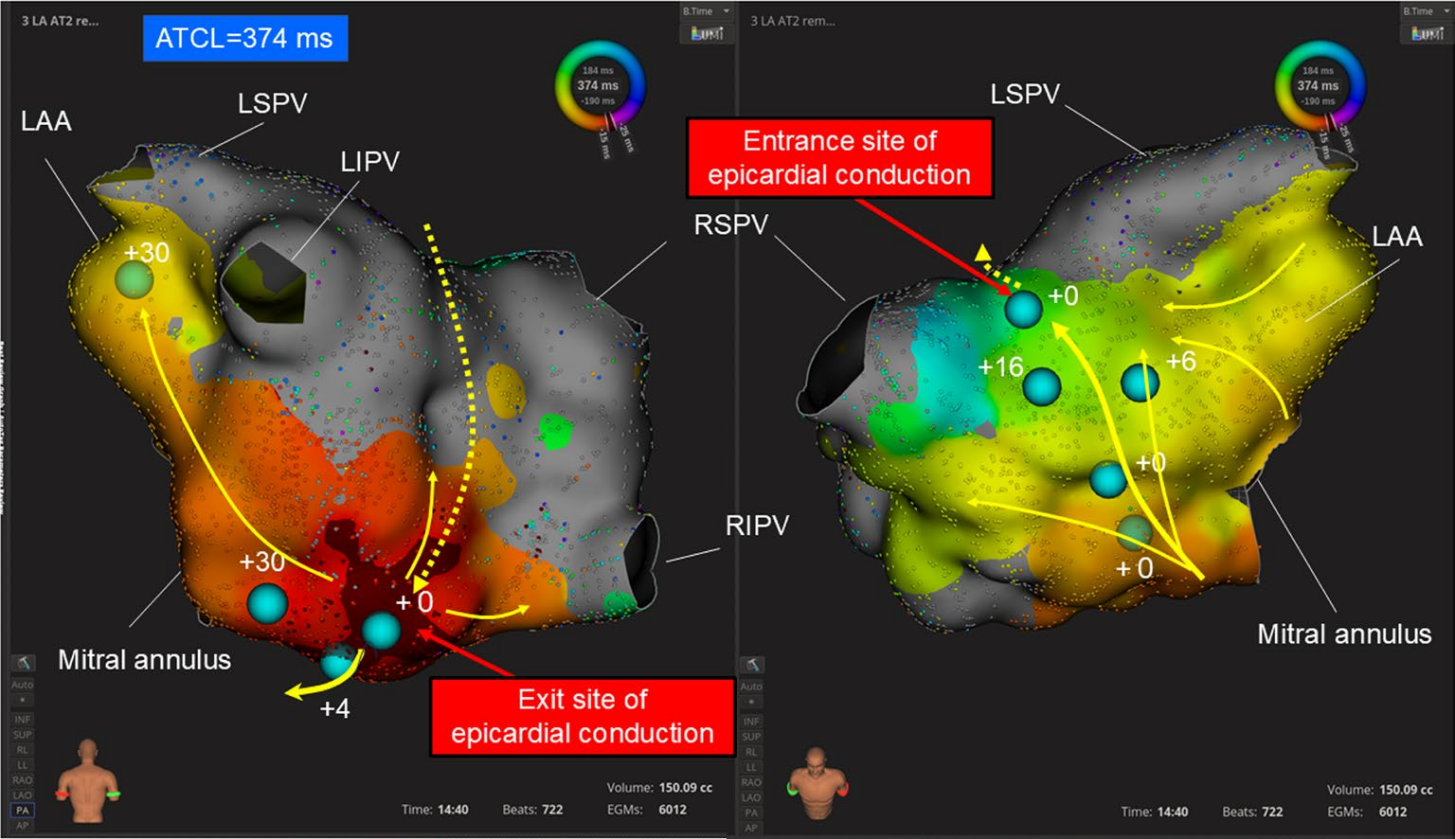
Activation map and entrainment findings in Patient 3. Light‐blue tags indicate the difference between PPI and ATCL, with values shown as “+xx (ms)”. Yellow arrows show endocardial activation of the AT. Based on entrainment findings, the possible epicardial channel of the AT is Septopulmonary bundle (yellow dashed arrow). Abbreviations as in Figure 1.

Patient 3 represented a typical case of a roof‐dependent AT involving the septopulmonary bundle (SPB) (Figure 3 and Video S3). The activation map initially suggested a focal AT originating near the center of the LA inferior wall, although the precise earliest activation site could not be clearly identified. Entrainment pacing from both the LA septum and anterior wall yielded PPI closely matching the ATCL, indicating a diagnosis of macroreentrant AT. The critical site—defined by a PPI equal to the ATCL—was located on the LA roof, suggesting that the SPB constituted part of the reentrant circuit and that the inferior wall functioned as a broad exit region. RF applications at the inferior exit site were ineffective; however, RF applications at the LA roof successfully terminated the AT within 10.8 s.

**FIGURE 4:**
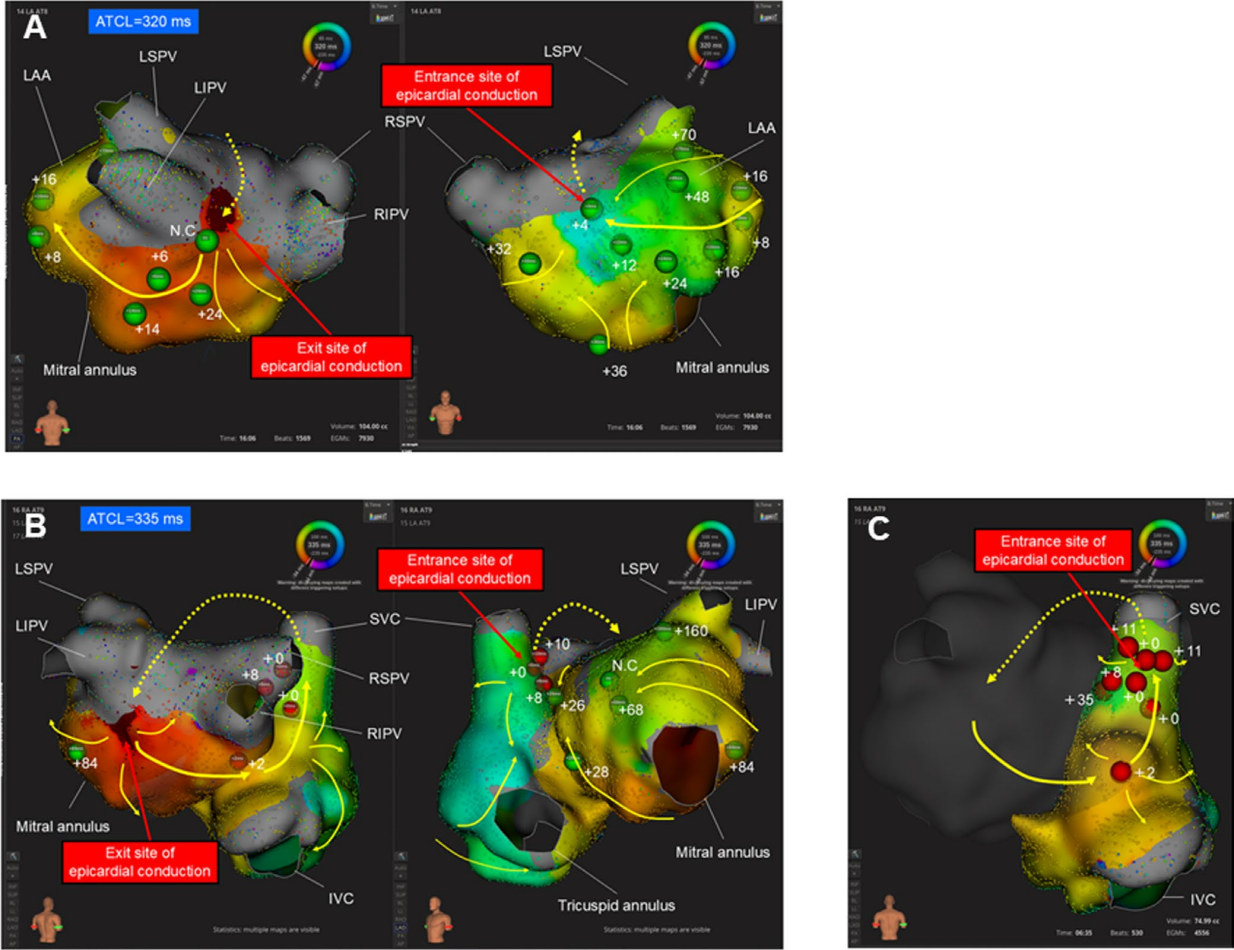
(A) Activation map and entrainment findings of AT1 in Patient 7. Green tags indicate the difference between PPI and ATCL, with values shown as “+xx (ms)”. Yellow arrows show endocardial activation of AT1. Based on entrainment findings, the possible epicardial channel of AT1 is Septopulmonary bundle (yellow dashed arrow). (B) Activation map and entrainment findings of AT2. Green and red tags indicate the difference between PPI and ATCL in LA and RA, respectively, with values shown as “+xx (ms)”. Based on entrainment findings, the possible epicardial channels of AT2 are Bachmann's bundle and Septopulmonary bundle (yellow dashed arrow). (C) Detailed entrainment findings of high RA in AT2. N.C = non‐capture; RA = right atrium; SVC = superior vena cava. Other abbreviations as in Figure 1.

Patient 7 exhibited the most complex ATs in our series. The activation map of AT1 suggested a focal AT originating near the center of the LA posterior wall (Figure 4A and Video S4). Entrainment pacing from the lateral MI, the bottom of the LAA, and the anterior wall yielded PPI closely matching the ATCL, consistent with a diagnosis of macroreentrant AT. The furthest downstream site where the PPI equaled the ATCL was located at the anterior edge of a scar on the LA roof. These findings suggested that AT1 was a roof‐dependent AT involving the SPB with the posterior wall serving as the exit region. After RF applications at the exit site failed to terminate the AT, we changed the target to the entrance at the anterior edge of the scar on the LA roof. RF applications there gradually prolonged the ATCL and ultimately led to the termination of AT1. However, atrial burst pacing reproducibly induced another AT (AT2), which exhibited a focal activation pattern with the earliest site located lower and more toward the left side of the posterior wall compared to AT1 (Figure 4B and Video S5). In the entrainment map, PPIs were significantly prolonged at the lateral MI, bottom of the LAA, and anterior wall compared to the ATCL, while the bottom of the right inferior pulmonary vein (RIPV) showed a PPI close to the ATCL. As the PPI was slightly prolonged at the LA septum, mapping of the right atrium (RA) was also performed, revealing a site where the PPI matched the ATCL. These findings suggested that AT2 was biatrial tachycardia (Bi‐AT) involving epicardial conduction via both the Bachmann bundle (BB) and the SPB. RF applications at the presumed exit site were interrupted due to esophageal temperature rise and failed AT termination. Detailed entrainment mapping at the posterior‐to‐septal aspect of the high RA identified the presumed entrance site of the BB (Figure 4C), and a total of four RF applications over a contiguous area at this region successfully terminated AT2. No further ATs were induced by atrial burst pacing.

The summary of the mapping and ablation results of ATs involving epicardial conduction is shown in Table 2. A total of 10 ATs related to epicardial conduction were observed in 9 patients. The ATCL was 328 ms (289–356). The missing activation time was 140 ms (93–157), corresponding to 42% (35–47) of the ATCL. Possible epicardial involvement was attributed to the VOM/LOM in four ATs, the SPB in another four, and combinations involving BB—specifically, BB with SPB in one AT and BB with VOM/LOM in another. While the EGM duration did not differ significantly between the exit and entrance sites (50 ms [42–66] vs. 40.5 ms [36–49], *p* = 0.23), the voltage at the entrance site was significantly higher than at the exit one (0.62 mV [0.27–0.79] vs. 0.20 mV [0.10–0.37], *p* = 0.014). In the analysis of EGM morphology, fragmented potential was more frequently observed at the exit sites (six of 10 ATs), whereas single potential was predominant at the entrance sites (six of 10 ATs). However, no significant association was observed in EGM morphology at the two sites (*p* = 1.00). In four patients, AT was terminated by the first RF application to the entrance site. In contrast, in the remaining five patients, including one with two ATs, immediate termination was not achieved. Additional RF applications were performed around the target site, resulting in progressive prolongation of the AT cycle length and eventual termination of the tachycardia. In all patients, the procedure was concluded after confirming non‐inducibility of ATs by atrial burst pacing.

**TABLE 2:** Mapping results of AT involving epicardial conduction.

Patient no.	ATCL	Missing activation time		Possible epicardial conduction	EGM at the exit site of epicardial conduction			EGM at the entrance site of epicardial conduction			Location of the entrance site	RF time to termination	Successful termination
	(ms)	(ms)	(%)		Duration (ms)	Voltage (mV)	Type	Duration (ms)	Voltage (mV)	Type		(sec.)	
1	289	145	50	VOM/LOM	52	0.25	SP	36	1.58	FP	Top of LAA ridge	4.7	Yes
2	356	134	37	VOM/LOM	30	0.42	FP	38	0.61	SP	Top of LAA ridge	25.3	Yes
3	374	165	44	SPB	66	0.15	FP	39	0.366	SP	LA roof	10.8	Yes
4	274	93	34	SPB	37	0.28	SP	29	0.689	FP	LA roof	76*	Yes
5	266	93	35	VOM/LOM	46	0.4	SP	49	0.233	SP	Top of LAA ridge	152**	Yes
6	366	200	55	VOM/LOM	69	0.1	FP	126	0.122	FP	Mid‐portion of LAA ridge	171***	Yes
7–1	320	150	47	SPB	64	0.1	FP	48	0.18	FP	LA roof	180**	Yes
7–2	335	135	40	SPB & BB	41	0.1	FP	17	5.3	SP	Septum portion of HRA	180**	Yes
8	344	157	46	BB & VOM/LOM	91	0.05	FP	59	0.83	SP	LA anterior wall	213***	Yes
9	313	65	21	SPB	48	0.53	SP	42	0.63	SP	LA roof	9.3	Yes

*Note:* RF time to termination represents the cumulative radiofrequency application time delivered to the entrance site until tachycardia termination.

Abbreviations: AT, atrial tachycardia; ATCL, atrial tachycardia cycle length; BB, Bachmann's bundle; EGM, electrogram; FP, Fragmented potential; LA, left atrium; LAA, left atrial appendage; LOM, ligament of Marshall; RF, radiofrequency; SP, single potential; SPB, septopulmonary bundle; VOM, vein of Marshall.

* 3 applications of RF.

** 4 applications of RF.

*** 5 applications of RF.

### 3.3 Complications

Patient 3, who was a 65‐year‐old woman with chronic kidney disease requiring hemodialysis, developed a stroke postoperatively, although MRI did not show a definitive infarction. Her symptoms completely resolved within approximately 48 h. In this case, ablation was performed using an open‐irrigated ThermoCool SF catheter (Biosense Webster, 56‐hole irrigation). RF energy was delivered at 35 W for approximately 35 s per application, with two at the exit site and three in the region of the presumed entrance site. No complications were observed in the other eight patients.

### 3.4 Follow‐Up

During a median follow‐up period of 198 days (150–342), four patients experienced recurrent atrial tachyarrhythmias, including two with AT, one with AF, and one with both AT and AF. Of these, three patients—excluding the patient with isolated AF recurrence—underwent repeat procedures. In the first (Patient 1), a typical peri‐mitral AT, which had not been observed during the initial procedure, was induced, whereas the index AT involving the VOM/LOM pathway was not observed. This AT was successfully treated with conventional endocardial lateral MI ablation combined with CS ablation. In the second (Patient 2), the clinical tachycardia was a roof‐dependent AT utilizing a gap in the previous roof line, and the previously treated peri‐mitral AT involving the VOM/LOM pathway was not inducible. Because recurrence of the index AT was initially suspected, mapping was also performed during pacing within the VOM; however, bidirectional block between the VOM and the LAA ridge was confirmed. In the third (Patient 6), recurrence of an AT involving the VOM/LOM pathway was observed and was successfully treated with ethanol infusion into the VOM. Overall, recurrence of the index AT involving the same epicardial conduction pathway was documented in only one patient.

## 4 Discussion

It is known that atrial myocardium comprises multiple layers of muscle fibers oriented in different directions, and that adipose tissue can be interposed between these layers [6, 7]. In addition, epicardial anatomical structures such as the CS and VOM/LOM, which bridge conduction pathways, have been identified in the region of the lateral MI [8, 9, 10].

Using high‐resolution mapping with multipolar catheters, several reports have described the endocardial mapping characteristics of AT involving epicardial conduction [2, 4]. These reports indicate that, on endocardial maps, the exit site of epicardial conduction may exhibit a pseudo‐focal pattern, and that findings such as the atrial global activation histogram are helpful for differentiating such ATs from true focal ATs.

Nakatani et al. reported that blocking the anatomical isthmus was more effective for AT termination than ablation at the exit site alone (20/21 [95%] vs. 1/5 [20%]) [4]. In contrast, Baskovski et al. achieved a 62.5% success rate in terminating AT by ablating the endocardial exit site [3]. In the present study, we retrospectively and systematically investigated cases in which AT was not terminated by initial exit‐site ablation, and then used entrainment mapping to approximate the location of the entrance site, which served as the next ablation target. While the study by Nakatani et al. demonstrated the limitations of targeting the endocardial exit site alone when epicardial conduction is involved, a finding that is consistent with our observations. Their approach primarily focused on creation of conduction block across an anatomical isthmus, whereas our approach focuses on identifying functionally relevant endocardial–epicardial connection sites using entrainment mapping. Importantly, these entrance sites are often located at the border of a previously ablated, densely scarred anatomical isthmus, suggesting that they represent a functional component within the same anatomical framework rather than a completely separate target. Ablation directed at these sites may therefore be viewed as a functionally guided refinement of anatomical ablation rather than an alternative strategy. By systematically applying this approach across diverse forms of epicardial conduction involving the VOM/LOM, SPB, and B.B.—including complex circuits where these pathways were combined—we provide a practical framework for effective ablation of challenging ATs. This strategy successfully terminated AT in all cases, confirming the clinical utility of the entrainment‐guided entrance site approach.

In some patients, the initial RF application to the entrance site failed to achieve immediate termination, and only gradual cycle‐length prolongation was observed. This progressive slowing of the tachycardia suggests that the epicardial conduction pathway was not confined to a discrete focal site but extended over a relatively broad region around the entrance. These findings indicate that, similar to the exit site, the entrance region may also be broad or composed of multiple connection fibers, requiring ablation over a wider area to achieve complete interruption. The eventual termination after additional ablation within this area implies that conduction delay accumulated as the ablation line expanded, ultimately interrupting the epicardial circuit.

While the exit site may appear focal on an endocardial activation map, it is generally impossible to identify the entrance site by endocardial mapping alone. Moreover, no previous studies have characterized the EGM features at the entrance site. In our small cohort, we were unable to identify consistent characteristics at the entrance site, except for a higher EGM amplitude compared to the exit site. Without epicardial mapping, detailed entrainment mapping—such as the approach used in our study—is currently the most reliable means of estimating the entrance site. With high‐resolution mapping using multipolar catheters now widely adopted, most two‐dimensional AT circuits can be delineated and the ablation target identified without entrainment mapping in many cases. Indeed, repeated entrainment risks terminating the AT, transitioning it to another AT, or degenerating it into atrial fibrillation. Entrainment has also been associated with a higher likelihood of altering the tachycardia than resetting it by a scanned extrastimulus [11]. Therefore, detailed entrainment mapping should be employed selectively to confirm the involvement of epicardial conduction in the AT circuit and to identify the location of the entrance site.

Recent studies have reported the use of pulsed field ablation (PFA) in the treatment of complex ATs [12, 13]. PFA has several advantages, including the absence of reported esophageal complications—eliminating the need for titration near the esophagus—and superior lesion formation in scarred myocardium, even across collagenous or fatty barriers, compared to RF ablation [14, 15]. The pentaspline and large‐tip catheters for PFA allow wide‐area ablation with fewer applications and reduced ablation time, potentially improving outcomes even for broadly connected exit sites of AT involving epicardial conduction [16]. Therefore, the somewhat complex stepwise strategy we employed in the present study may no longer be necessary when performing PFA. Further studies are warranted to evaluate the efficacy of PFA in ATs involving epicardial conduction.

In our series, recurrence of the index epicardial AT was uncommon, and most late atrial tachyarrhythmias represented different ATs or AF rather than recurrence of the originally treated circuit, which may in part be attributable to several procedural factors. First, detailed entrainment mapping enabled precise characterization of the complex reentrant circuit and localization of the endocardial–epicardial connection corresponding to the entrance site. Rather than delivering RF applications within dense scar along the anatomical isthmus, ablation was directed to the endocardial aspect of these connection sites, typically located at the border of the scar, where interruption of the pathway could be achieved more effectively. Second, careful induction testing using atrial burst pacing was systematically performed after termination of the tachycardia to confirm non‐inducibility before completion of the procedure. This may be particularly important because conduction through these pathways may be slow and occur along the border of scarred tissue, making confirmation of complete block by simple pacing maneuvers potentially unreliable.

## 5 Study Limitations

This study has several limitations. First, patients were retrospectively selected based on the performance of entrance‐site ablation, and detailed entrainment mapping was not systematically performed in all AT cases during the study period. Consequently, some epicardial ATs may have been inadvertently classified as focal tachycardias and successfully terminated by exit‐site ablation without comprehensive entrainment evaluation. Therefore, the true incidence of AT involving epicardial conduction and the proportion of cases requiring an entrance‐site approach cannot be determined from this study. Second, orthodromic capture of the exit site from the defined entrance site was not systematically assessed in all cases, which may limit definitive confirmation of the entrance location. Furthermore, assessment of pathway interruption after successful ablation was based on confirmation of disappearance of breakout at the previously identified exit site during pacing from sites adjacent to the presumed entrance region. Evaluation of conduction in the opposite direction was not systematically performed, and therefore bidirectional functional interruption of the presumed epicardial pathway could not be confirmed. Third, of the 10 ATs presented, five ATs were diagnosed as VOM/LOM‐related AT based solely on endocardial activation mapping and entrainment findings. In many reports on VOM/LOM‐related AT, a very thin electrode catheter is inserted into VOM to confirm the diagnosis; however, this was not performed in any of our cases [17]. Furthermore, ethanol infusion into the VOM has been reported to effectively prevent recurrence in VOM/LOM‐related AT [10, 18]. In fact, one patient in our series underwent ethanol infusion into the VOM during a redo procedure, suggesting that ethanol infusion may have been preferable as a first‐line treatment. Nevertheless, because a suitable VOM for ethanol infusion cannot be identified in approximately 16% of cases, knowledge of our study's approach remains clinically important [19]. In our clinical practice, an entrainment‐guided entrance‐site ablation is typically attempted when exit‐site ablation fails, and VOM ethanol infusion is considered as an alternative or adjunctive strategy depending on anatomical feasibility and procedural context.

## 6 Conclusion

In patients with AT involving epicardial conduction, a systematic entrainment‐guided approach to identify and ablate the entrance site proved effective when conventional exit‐site ablation was insufficient. This strategy may serve as a practical framework for managing complex AT circuits mediated by multiple epicardial connections.

## Funding

The authors received no specific funding for this article.

## Ethics Statement

This study was approved by the Institutional Review Board of Toyohashi Heart Center (Approval No. 250701). This study was conducted in accordance with the ethical principles outlined in the Declaration of Helsinki. All authors confirm that the research adheres to the ethical guidelines of the Journal of Arrhythmia and follows the established standards of research integrity, including proper citation and avoidance of plagiarism.

## Consent

Informed consent was obtained from all participants.

## Conflicts of Interest

Yuichiro Sakamoto has received lecture fees from Abbott, Boston Scientific, Biosense Webster, and Medtronic. He has also served as an advisory board member for Boston Scientific, Biosense Webster, and Abbott (each one‐time participation). Daisuke Yoshimoto has received a lecture fee from Medtronic. Other authors have no conflicts.

## Supporting information


**Video S1:** Activation map in Patient 1.

**Video S2:** Activation map in Patient 2.

**Video S3:** Activation map in Patient 3.

**Video S4:** Activation map of AT1 in Patient 7.

**Video S5:** Activation map of AT2 in Patient 7.

## Data Availability

The data that support the findings of this study are available on request from the corresponding author. The data are not publicly available due to privacy or ethical restrictions.
